# Building up resources and knowledge to unravel transcriptomics dynamics underlying *Eucalyptus globulus* xylogenesis

**DOI:** 10.1186/1753-6561-5-S7-O52

**Published:** 2011-09-13

**Authors:** Jorge AP Paiva, José Carlos Rodrigues, Pedro Fevereiro, Lucinda Neves, Clara Araújo, Cristina Marques, Ana Teresa Freitas, Hélène Bergès, Jacqueline Grima-Pettenati

**Affiliations:** 1 Instituto de Investigação Científica e Tropical (IICT/MCTES) Palácio Burnay - Rua da Junqueira, 30, 1349-007 Lisboa, Portugal Instituto de Biologia Experimental e Tecnológica (IBET) Av. da República, Quinta do Marquês, 2781-901 Oeiras, Portugal; 2 Instituto de Investigação Científica e Tropical (IICT/MCTES) Palácio Burnay - Rua da Junqueira, 30, 1349-007 Lisboa, Portugal; 3 Instituto de Biologia Experimental e Tecnológica (IBET) Av. da República, Quinta do Marquês, 2781-901 Oeiras, Portugal; 4 ALTRI FLORESTAL SA, Quinta do Furadouro 2250-582 Olho Marinho, Portugal; 5 RAIZ, Herdade de Espirra, 2985-270 Pegões, Portugal; 6 INESC-ID - Instituto de Engenharia de Sistemas e Computadores, R. Alves Redol 9, 1000 Lisbon, Portugal; 7 INRA-CNRGV - chemin de Borde Rouge 31326 Castanet-Tolosan, France; 8 UMR CNRS/Université Toulouse III 5546, Pôle de Biotechnologies Végétales, 24 chemin de Borde Rouge, BP42617 Auzeville, 31326 Castanet, Tolosan, France

## 

The economic importance of some *Eucalyptus* species, including interspecific hybrids, has been extended from the traditional interest of pulp and paper production to the emergent areas of bio-fuels and bio-materials. New genomic resources and high throughput technologies have provided the *Eucalyptus* research international community with the opportunities to identify genomic regions of interest in order to comprehensively dissect, catalogue and characterize genes involved in the determination of wood formation and quality. Similar strategies can be now applied to identify key regulator genes and better understand the cellular mechanisms by which they modulate the complex molecular events occurring in xylogenesis.

The *Geneglob^wq^* project (2006-2010) produced and used a set of genomic tools which, associated with Next Generation Sequencing (NGS) technologies, allowed us to expand the knowledge of the *Eucalyptus* genome by focusing on regions potentially involved in the determination of wood properties, namely pulp yield and lignin content. We first start to characterize two *E. grandis* BAC libraries [1] constructed by the Arizona Genome Institute (with DNA from the clone Brasuz S1 whose genome was sequenced recently by the DOE [http://www.jgi.doe.gov/sequencing/why/99176.html], and two *E. globulus* BAC libraries made available by RAIZ [http://www.raiz-iifp.pt/]. We than used 3D-pools of BAC libraries and BAC macroarrays to characterize genomic environment of several lignin and lignin-regulator genes (e.g.*EguCCR*, *EguCAD2* and *EguRAC1*) both in *E. grandis* and *E. globulus*. The shotgun sequencing of selected BAC clones containing those genes generated a high amount of sequencing data that made it possible to map the *E. globulus* BAC sequenced clones against the *E. grandis* genome (8X coverage). These comparative analyses showed extended microlinearity between both genomes, at least in the studied regions. Additionally, we have sequenced and annotated the chloroplast genome of *E. grandis* (GeneBank Accession NC_014570) [1].

A global approach to unravel *E. globulus* transcriptome dynamics has also been included and structured in the *Geneglob^wq^* project, aiming at the identification of genomic hotspots of transcription activity. Various *E. globulus* xylogenesis “models” have been considered comprising several paired, contrasting wood forming tissues (provided by RAIZ): i) xylem samples collected along the year (season variation); ii) juvenile and adult individuals of a single genotype; iii) contrasting genotypes for pulp yield. Samples from these tissues were used for transcriptome sequencing using *IlluminaHi-Seq* technology (*mRNA-SEQ*). The same *E. globulus* genotype used for both *E. globulus* BAC libraries (a parent tree used in controlled crosses by RAIZ) has been re-sequenced (pair-end 100bp), and provided the first draft of an *E. globulus* genome. This resequencing data was mapped against the *E. grandis* Brasuz S1 reference genome. Transcriptomic data were also blatted against the gene models annotated in *E. grandis* genome, to evaluate *in silico* the expression of each gene.

More recently, the *microEGo* project (2010-2012) started the identification and characterization of *Eucalyptus globulus* microRNAs and their target genes, involved in the regulation of wood formation. The *E. globulus* season variation xylogenesis “model” was used considered for this project as well as an *E. globulus* reaction wood “model”. The latter comprises reaction wood tissues (tension / opposite wood) formed in bent trees at different kinetic times of gravitropic stimulation and control wood (non-bent trees). Small RNA libraries have been generated from those tissues and sequenced using *IlluminaHi-Seq* technology (*Small RNAs-SEQ*). The sequencing data from both *microEGo* and *Geneglob^wq^* projects together with a genome wide bioinformatics analysis of *E. grandis* reference genome and *E. globulus* genome are being used for identification of miRNA gene and putative their putative target-genes.

These projects will hopefully constitute an important piece in the assemblage of whole new categories of knowledge and genomic resources for the *Eucalyptus* community, providing insights into the nature of the molecular machinery involved in wood formation and most importantly in the identification of key players determining the variability of wood characteristics and its end-uses.
